# An Integrated Model of Multiple-Condition ChIP-Seq Data Reveals Predeterminants of Cdx2 Binding

**DOI:** 10.1371/journal.pcbi.1003501

**Published:** 2014-03-27

**Authors:** Shaun Mahony, Matthew D. Edwards, Esteban O. Mazzoni, Richard I. Sherwood, Akshay Kakumanu, Carolyn A. Morrison, Hynek Wichterle, David K. Gifford

**Affiliations:** 1 Center for Eukaryotic Gene Regulation, Department of Biochemistry & Molecular Biology, The Pennsylvania State University, University Park, Pennsylvania, United States of America; 2 Computer Science and Artificial Intelligence Laboratory, Massachusetts Institute of Technology, Cambridge, Massachusetts, United States of America; 3 Department of Biology, New York University, New York, New York, United States of America; 4 Division of Genetics, Department of Medicine, Brigham and Women's Hospital and Harvard Medical School, Boston, Massachusetts, United States of America; 5 Departments of Pathology and Cell Biology, Neurology, and Neuroscience, Center for Motor Neuron Biology and Disease, Columbia Stem Cell Initiative, Columbia University Medical Center, New York, New York, United States of America; Ottawa University, Canada

## Abstract

Regulatory proteins can bind to different sets of genomic targets in various cell types or conditions. To reliably characterize such condition-specific regulatory binding we introduce MultiGPS, an integrated machine learning approach for the analysis of multiple related ChIP-seq experiments. MultiGPS is based on a generalized Expectation Maximization framework that shares information across multiple experiments for binding event discovery. We demonstrate that our framework enables the simultaneous modeling of sparse condition-specific binding changes, sequence dependence, and replicate-specific noise sources. MultiGPS encourages consistency in reported binding event locations across multiple-condition ChIP-seq datasets and provides accurate estimation of ChIP enrichment levels at each event. MultiGPS's multi-experiment modeling approach thus provides a reliable platform for detecting differential binding enrichment across experimental conditions. We demonstrate the advantages of MultiGPS with an analysis of Cdx2 binding in three distinct developmental contexts. By accurately characterizing condition-specific Cdx2 binding, MultiGPS enables novel insight into the mechanistic basis of Cdx2 site selectivity. Specifically, the condition-specific Cdx2 sites characterized by MultiGPS are highly associated with pre-existing genomic context, suggesting that such sites are pre-determined by cell-specific regulatory architecture. However, MultiGPS-defined condition-independent sites are not predicted by pre-existing regulatory signals, suggesting that Cdx2 can bind to a subset of locations regardless of genomic environment. A summary of this paper appears in the proceedings of the RECOMB 2014 conference, April 2–5.

## Author Summary

Many proteins that regulate the activity of other genes do so by attaching to the genome at specific binding sites. The locations that a given regulatory protein will bind, and the strength or frequency of such binding at an individual location, can vary depending on the cell type. We can profile the locations that a protein binds in a particular cell type using an experimental method called ChIP-seq, followed by computational interpretation of the data. However, since the experimental data are typically noisy, it is often difficult to compare the computational analyses of ChIP-seq data across multiple experiments in order to understand any differences in binding that may occur in different cell types. In this paper, we present a new computational method named MultiGPS for simultaneously analyzing multiple related ChIP-seq experiments in an integrated manner. By analyzing all the data together in an appropriate way, we can gain a more accurate picture of where the profiled protein is binding to the genome, and we can more easily and reliably detect differences in protein binding across cell types. We demonstrate the MultiGPS software using a new analysis of the regulatory protein Cdx2 in three different developmental cell types.

This article is associated with RECOMB 2014.

## Introduction

Profiling the activity of regulatory proteins in multiple cell types is important for understanding cellular function, as a single regulator can bind to distinct sets of genomic targets depending on the cellular context in which it is expressed. Characterizing the determinants of such binding specificity is key to understanding how a single regulator can play multiple roles during development and other dynamic cellular processes. For example, pre-existing genomic context such as chromatin accessibility or the binding of other regulators may determine the binding of some developmental transcription factors (TFs) [1]–[3], while other ‘pioneer’ TFs may find their binding targets independently of the established chromatin state [4], [5].

Here we introduce MultiGPS, an integrated machine learning approach for the analysis of condition-specific binding events from multi-condition ChIP-seq data. MultiGPS performs binding event analysis across multiple conditions, sharing information across conditions to produce accurate joint binding estimates while simultaneously allowing for condition-specific binding events. MultiGPS employs a flexible framework for incorporating prior information into binding event discovery, allowing models of joint binding and sequence dependence to be used. The novel multi-experiment modeling approach of MultiGPS identifies the read enrichment associated with binding events that are bound in specific conditions, enabling principled methods of discovering differential binding [6]–[9].

Most current strategies for defining consistent ChIP-seq binding event locations across multiple experiments either analyze each experiment independently or pool reads for analysis. For example, the ENCODE2 project used standard ChIP-seq event finders on each experiment independently, and then merged event locations across experiments using a fixed-sized window to define event identity [10], [11]. Related methods specifically developed for multi-condition ChIP-seq analysis require that binding events be called in each condition individually as a preprocessing step, then apply statistical models to matched regions to detect differential effects [9], [12]. Other multi-condition approaches focus on ChIP-seq signals arising from broad regions of enrichment, such as histone modifications. These methods instead search for larger genomic regions where coverage patterns differ across experiments [8], [13]–[15]. In contrast, MultiGPS uses a joint multi-experiment model that considers the read data from all experiments to produce accurate location estimates of punctate binding events.

Approaches that first identify binding events and then attempt to merge locations across conditions may inappropriately combine distinct binding events that happen to be located within the same window. In genomic regions with a high density of binding events, the problem of matching sites across conditions is difficult and may lead to erroneous comparisons between binding strengths. Furthermore, the experiment-by-experiment event calling approach fails to use the full power of the experimental data when a large fraction of binding events are shared across conditions. An alternative method is to pool ChIP-seq reads from all experiments and then use a single event finding run to yield a consistent set of binding event locations that can be subsequently quantified in each individual experiment. However, this pooling approach may not discover weak condition-specific binding locations that are swamped by noise from other experiments in the pooled set of reads. Additionally, applying a single detection threshold in the pooled read set may bias the binding event calls to experiments that had higher sequencing coverage, better antibody batches, or fewer technical sources of error. Similarly, varying experimental parameters such as the fragmentation distribution could render the pooled read dataset harder to analyze by algorithms that assume a single, consistent set of experimental properties.

MultiGPS combines the theoretical benefits of pooling and separate ChIP-seq experimental analysis by using a Bayesian prior to couple the analysis of independent experiments together. This multi-experiment model is one aspect of a novel modeling approach that enables external sources of information to be included as priors in binding event identification (see Methods). In this work, we use the following priors, while recognizing that other directions are also possible:

- A sparsity-promoting prior on binding event strengths to encourage a core set of statistically-significant sites while allowing for closely-spaced binding event deconvolution (as in [16]).
- A genome sequence-based (motif) prior to allow binding events to express a sequence preference, particularly useful for automatically aligning sites to confident motif hits in the small region implicated by the read data around a binding event (similar to [17]).
- An inter-experiment prior that encourages location coherence across experimental conditions, allowing for more effective joint experiment analysis and automatic data-guided event alignment.

MultiGPS detects binding events independently in each experiment in each step of its iterative optimization, allowing it to model experiment-specific parameters such as the distribution of reads around binding events and the properties of background noise. The iterative optimization procedure analyzes each experimental condition in turn, using binding event locations from other experiments to form an inter-experiment prior term for a single experiment optimization. MultiGPS therefore encourages the base locations of binding events to align across experiments when appropriate, and automatically produces coherent sets of binding events that are linked across experiments without any potentially noisy windowed analysis. To our knowledge, MultiGPS is the first ChIP-seq analysis approach that uses read data from multiple experiments in a joint and fully integrated method for identifying consistent and accurate binding event locations.

As a case study of our framework's sensitive and accurate multi-condition analysis, we applied MultiGPS to Cdx2 binding data in three developmentally relevant cellular contexts and found that condition-specific Cdx2 binding events are predicted by preexisting chromatin state. Surprisingly, condition-independent Cdx2 binding events that are bound in multiple contexts do not appear to be predetermined by accessibility or other chromatin signatures, and instead may be predicted on the basis of cognate motif occurrence. Our results suggest that Cdx2 can act as a pioneer factor at a subset of sites, while also being influenced by preexisting genomic context at other sites. Therefore, our results have consequences for understanding where TFs will bind when introduced into an established regulatory state during development, or when induced artificially during cellular programming techniques.

## Results

### MultiGPS encourages consistent binding event locations across experiments

**Figure 1:**
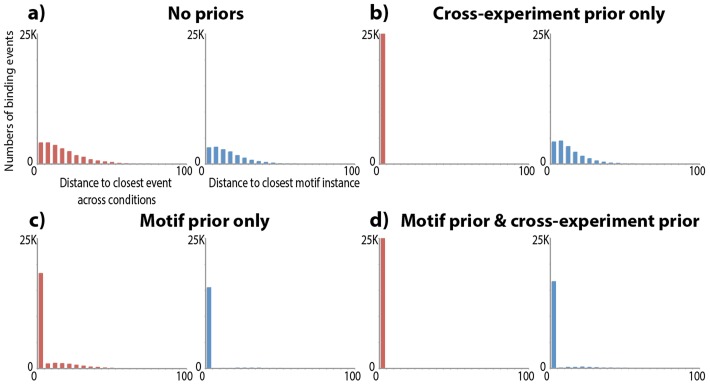
MultiGPS binding event consistency with or without the use of motif and inter-experiment priors. MultiGPS was run on two ENCODE GM12878 CTCF ChIP-seq biological replicate experiments, treating them as distinct conditions. Red histograms show the distance in base pairs from each predicted binding event in one experiment to the nearest binding event in the other condition (if within 100 bp). Blue histograms show the distance from each binding event to the nearest strong match to the CTCF motif (if within 100 bp).

We find that MultiGPS's inter-experiment and motif priors encourage binding location consistency on CTCF biological replicate experiments. The binding events that are called in both CTCF replicates should by definition be located at the same base location. As we can see in Figure 1a, when MultiGPS is run without either prior, predicted binding events do not typically align to each other or to cognate motif instances. Each prior alone makes a significant, though incomplete, improvement in binding event accuracy (Figure 1b–c). The inter-experiment prior is able to significantly improve the distance to the nearest motif when compared to sites identified without any positional priors (*p*<5×10^−5^, Mann-Whitney *U* test comparing binned distance to nearest motif match). The motif prior significantly improves the distance to the nearest site in another experiment (*p*<1×10^−12^, Mann-Whitney *U* test comparing binned distance to the nearest event in another experiment). In these two comparisons, we used information sources not considered by the prior as validation (motif distance for the inter-experiment prior and inter-experiment distance for the motif prior). The use of both priors together fully utilizes available sequence and multi-experiment information and allows almost all binding events in this example to be aligned to consistent (typically motif-associated) locations (Figure 1d). These comparisons are not meant as absolute performance assessments for the MultiGPS modeling approach, but instead as relative measurements of the benefit of using additional types of prior information within a single modeling framework.

### MultiGPS outperforms alternative approaches in simulated multi-condition ChIP-seq datasets

**Figure 2:**
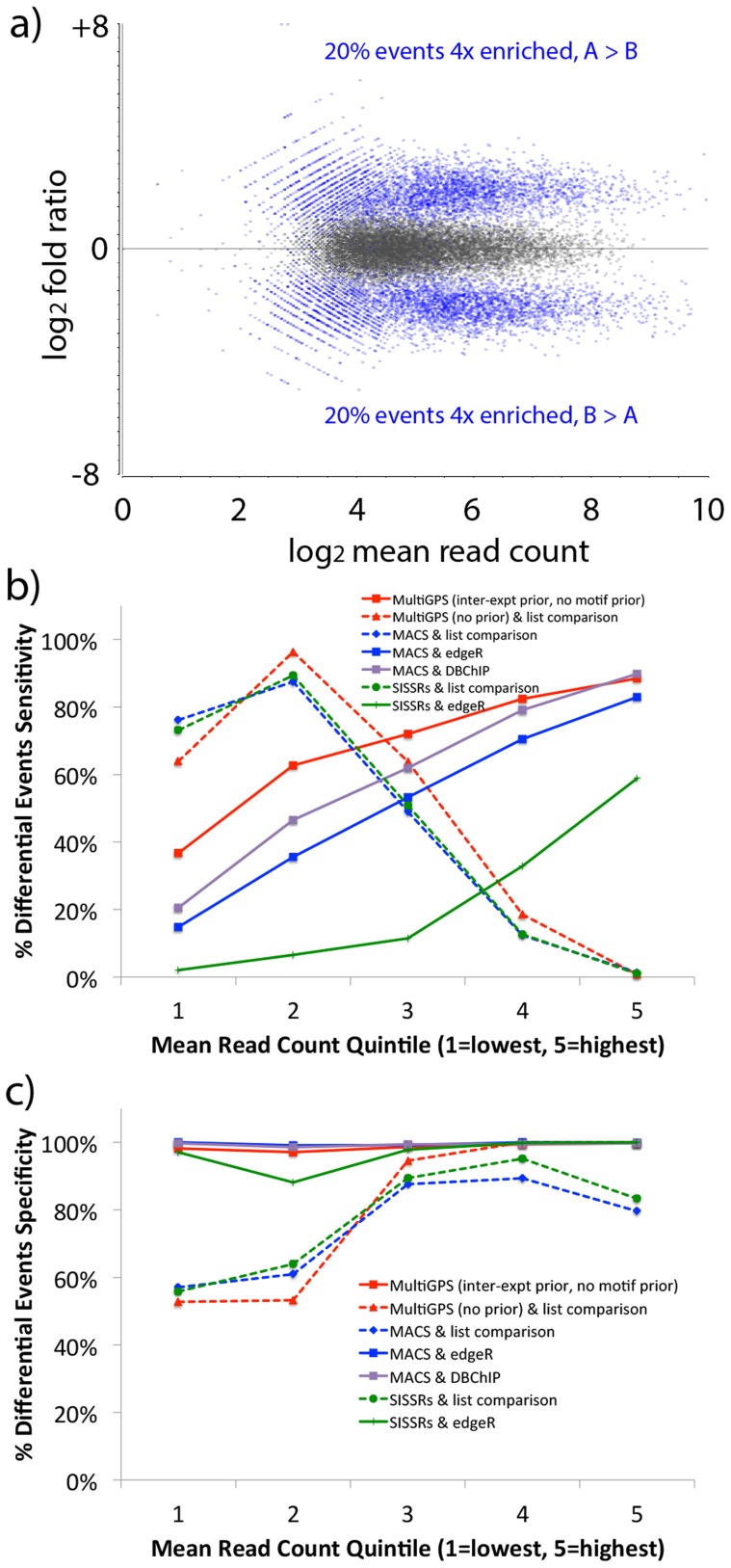
a) MA plot displaying the mean read count and log fold ratio distributions of the simulated ChIP-seq dataset in which 40% of binding events are defined as 4-fold differentially enriched in one condition versus the other. Defined differential events highlighted in blue, non-differential in gray. **b)** Sensitivity and **c)** specificity of various approaches when predicting differentially bound events. Results are broken out by quintile on the mean read count across conditions (i.e. based on x-axis in **a)**).

MultiGPS facilitates the detection of differential binding events by accurately quantifying read count levels associated with each binding event in each analyzed experiment. Since at present no ChIP-seq datasets exist for which absolute binding levels are known across multiple conditions, we generated simulated ChIP-seq datasets to test the relative performance of MultiGPS in defining differential binding events. In our simulated data, the distribution of reads at binding events mirrors the properties of real ChIP-seq datasets (see Methods). A subset of binding events is chosen to be differentially enriched across conditions, and while we chose to set the absolute level of differential enrichment to be constant at all differential events (4-fold in Figure 2, 8-fold in Figure S1), simulated sampling noise leads to a wide array of apparent fold differences (Figure 2a, blue dots).

Using the simulated data, we compared MultiGPS with other approaches for determining differential binding events. We used MultiGPS (without the motif prior since no sequence information was used to simulate the data), MultiGPS in single-condition mode (i.e. without using either inter-experiment or motif priors), and the single-condition event finders MACS [18] and SISSRs [19] to predict binding events in each simulated condition. All methods made comparable numbers of binding event predictions in each dataset (Figure S2). For the methods other than MultiGPS, differential binding events were defined using: a) binding event list comparison, where differential binding events are those that are detected in one condition and no binding event is detected within 200 bp in the other condition; b) using the software DBChIP [9]; or c) by counting reads that occur within the enriched regions and inputting the resulting tables into edgeR [6] (using the same parameters as used by edgeR within MultiGPS).

The results illustrate the problems with defining differentially bound events using binding event list comparison. Regardless of which event finding method was used to provide input binding events, list comparisons have poor sensitivity when predicting differentially bound events with higher mean read counts (Figure 2b, dashed lines). Such events are more likely to be detected in both conditions and hence would be treated as non-differential binding events regardless of quantitative differences in ChIP enrichment levels. Conversely, binding event list comparisons have low specificity when predicting differentially bound events with lower mean read counts (Figure 2c, dashed lines). Low enrichment binding events may have read counts that are just above a binding event detection threshold in one condition, and just below in another, even if there is no significant quantitative difference in the underlying ChIP enrichment levels. Such events would appear as false positive differential binding event predictions according to the binding event list comparison approach.

In contrast, approaches that test differential binding using statistical analyses of read count tables have uniformly high specificity across our test datasets (Figure 2c, solid lines). These methods also have higher sensitivity when predicting differential binding events with higher mean read counts (Figure 2b, solid lines) or involving greater absolute differences in binding levels (Figure S1b, solid lines). EdgeR attains the highest overall sensitivity using the read count tables generated by MultiGPS, thus illustrating the advantages of MultiGPS' probabilistic approach to quantifying read enrichment at binding sites across conditions.

### MultiGPS improves the quantification of condition-specific binding events in ENCODE data

MultiGPS models experiment-specific parameters such as the distribution of reads around binding events and the properties of background noise. To investigate whether these parameters yield improved quantification of binding event ChIP enrichment, we ran the complete MultiGPS model on 14 ChIP-seq experiment sets in which the ENCODE2 project has performed replicated ChIP-seq of a given protein in all three human Tier 1 cell lines. While no gold standard exists for measuring the accuracy of ChIP-enrichment quantification, we reasoned that accurate quantification estimates should be correlated across biological replicate experiments. For each of the 14 experiment sets, MultiGPS yields per-replicate estimates of binding enrichment for binding events discovered in any cell line. We compared these values to those produced by the widely used approaches of counting read occurrences in a window around the binding event locations (here we use a 400 bp window centered on the MultiGPS-defined binding event locations), or by using the peak heights defined by MACS [18] analyses of the same data. Quantified read counts were compared across biological replicate pairs using Spearman's rank correlation, a nonparametric assessment of statistical dependence that makes no distributional assumptions that could artificially favor one model over another. Note that MACS does not produce per-replicate read counts or peak heights at each event, and so to compare MultiGPS with MACS we ran MACS on each replicate separately and compared read counts and heights at only those binding events detected in both replicates by MACS and MultiGPS. Read counts at these reproducibly detected binding events may be more highly correlated than read counts associated with the wider sets of binding events tested in the comparison between MultiGPS estimates and windowed read counts.

**Figure 3:**
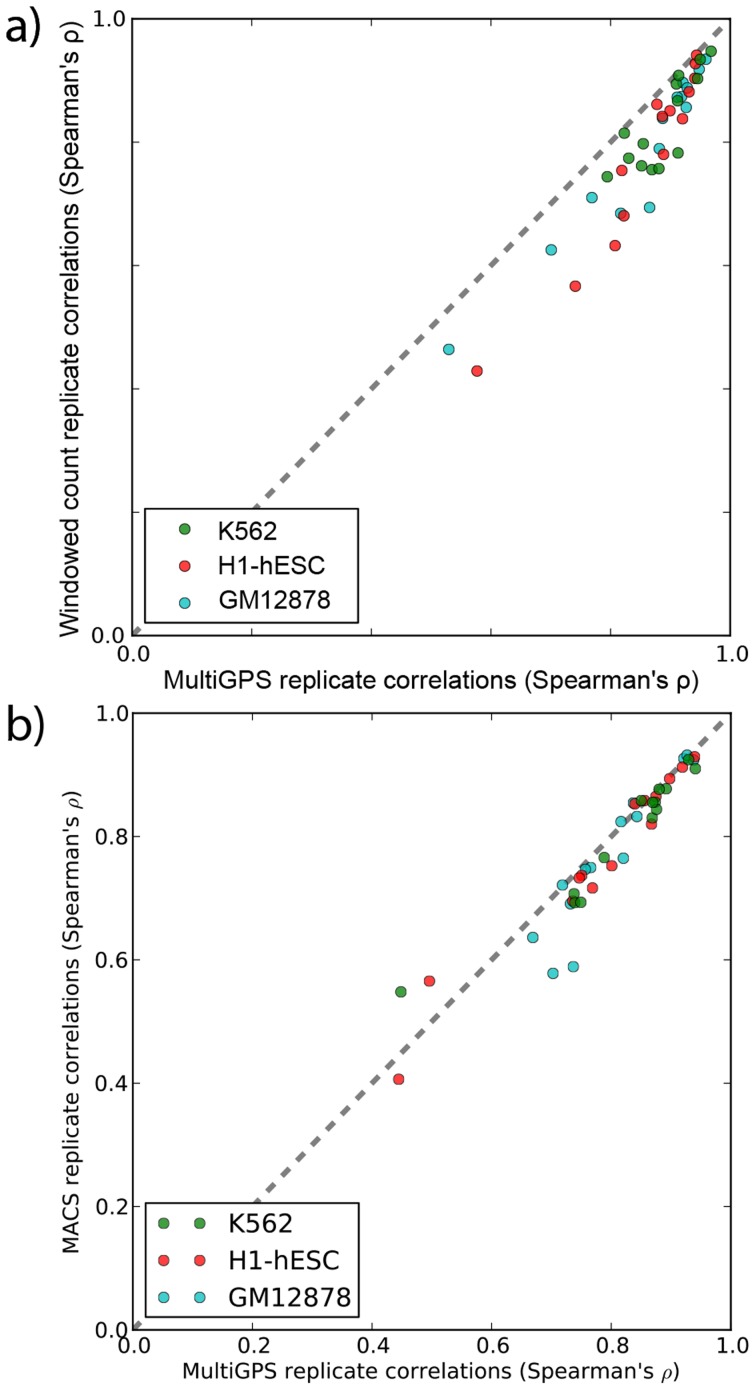
MultiGPS probabilistic ChIP-enrichment estimates improve binding site quantification consistency across replicates. MultiGPS was used to identify binding sites on 14 ENCODE transcription factor ChIP-seq experiments in three Tier 1 cell types. Binding site strengths were quantified with MultiGPS's probabilistic model, incorporating an integrated noise model and read sharing among nearby binding sites, and either: **a)** windowed counts, which sum up reads in a 400 bp window centered on the MultiGPS predicted binding location; or **b)** peak heights defined by MACS. MultiGPS produces more replicable quantifications for all examined factors in **a)**, and most in **b)**.

As shown in Figure 3, MultiGPS improves the cross-replicate correlation of binding event quantification estimates in most tested datasets, implying that MultiGPS has reduced the effects of inter-replicate noise in comparison to the window counting approaches. We expect that reducing the degree of over-dispersion between replicates will yield greater sensitivity in detecting significant differences between conditions. Indeed, in all 14 tested datasets we find substantially greater numbers of statistically significant differentially enriched binding events between cell lines when we run edgeR [6] on the MultiGPS quantification table as opposed to the table of read counts produced by the window approach (Table S1). Therefore, MultiGPS improves the quantification of binding event ChIP-enrichment and the detection of condition-specific binding events.

### Condition-specific Cdx2 binding event detection is improved by MultiGPS

To demonstrate the ability of MultiGPS to analyze biologically relevant condition-specific binding events, we examined if MultiGPS improves upon the independent analysis of experiments when identifying Cdx2 events in multiple conditions. Cdx2 is a mammalian caudal-type homeobox protein that plays a key role in regulating the development of diverse tissue types. For example, Cdx2 is a master regulator of the intestinal lineage when expressed in endoderm [20], and also plays a key role in defining caudal motor neuron fate when expressed in motor neuron progenitors (pMNs) [21]. In addition, over-expression of Cdx2 in embryonic stem (ES) cells forces cells to differentiate into the trophectoderm lineage [22], [23]. We thus wanted to elucidate how Cdx2 performs its different regulatory functions in these three developmental contexts. Does it bind to the same genomic targets in all cell types, or does it bind distinct targets in each context? If the latter, how is such specificity achieved? To determine the context-dependent binding activity of Cdx2, we performed ChIP-seq analysis of Cdx2 after it was over-expressed in ES cells, endoderm, and pMNs. We call these cell types after Doxycycline-dependent Cdx2 induction ES+Dox Cdx2, endoderm+Dox Cdx2, and pMN+Dox Cdx2, respectively. Since Cdx2 is not natively expressed in any of these three cell types, our experiments provide a useful model of how a transcription factor responds to a new cellular environment.

**Figure 4:**
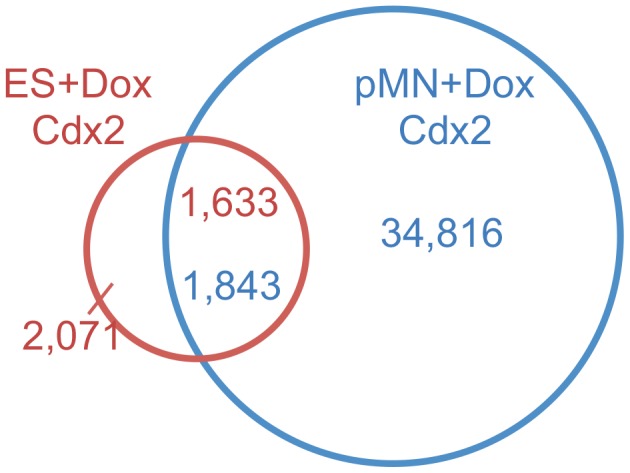
Binding event list comparison approaches to differential binding overestimate differences between binding site lists due to threshold effects and ignore true differences in enrichment between sites that are called bound in multiple conditions. The Venn diagram shows the overlap between ES+Dox and pMN+Dox binding events using a 200 bp overlap window. 1,633 ES+Dox Cdx2 events are within 200 bp of pMN+Dox events, while 1,843 pMN+Dox events are within 200 bp of ES+Dox events.

We found that MultiGPS outperformed an independent binding event analysis (i.e. using independent runs of MultiGPS without the use of priors) on the three Cdx2 conditions using a binding event list comparison approach to determine differentially bound sites. While this is a common approach in the literature, it leads to highly misleading results. As can be seen in Figure 4, the binding event list comparison suggests that 95% of pMN+Dox sites are not bound in ES+Dox cells. However, the apparent degree of differential binding is largely caused by the disparity in the total numbers of binding events predicted in each condition (3,704 in ES+Dox and 36,651 in pMN+Dox). The difference in the total number of events is in turn caused by differences in read coverage between the conditions and the thresholds employed to determine bound events. In addition, the binding event list comparison approach may miss differences at events when the level of ChIP enrichment varies significantly between conditions. To perform a more principled analysis of Cdx2 differential binding, we analyzed the ChIP-seq data collection using MultiGPS (Table 1). With the coupled MultiGPS method only 24% of all pMN+Dox Cdx2 binding events are significantly differentially enriched in pMN+Dox cells compared with ES+Dox cells (*p*<10^−3^), while 37% of all ES+Dox Cdx2 binding events are significantly differentially enriched in ES+Dox cells compared with pMN+Dox (*p*<10^−3^).

**Table 1:** Numbers of significantly enriched Cdx2 binding events called by MultiGPS in each condition.

	Total Significant Events	>ES+Dox (*p*<10^−3^)	>pMN+Dox (*p*<10^−3^)	>Endo+Dox (*p*<10^−3^)
ES+Dox	4,581	/	37%	31%
pMN+Dox	38,423	24%	/	8%
Endo+Dox	35,394	49%	22%	/

Percentages of called events that are significantly enriched in one condition over another are also shown.

### Condition-specific Cdx2 binding is predetermined by genomic context

**Figure 5:**
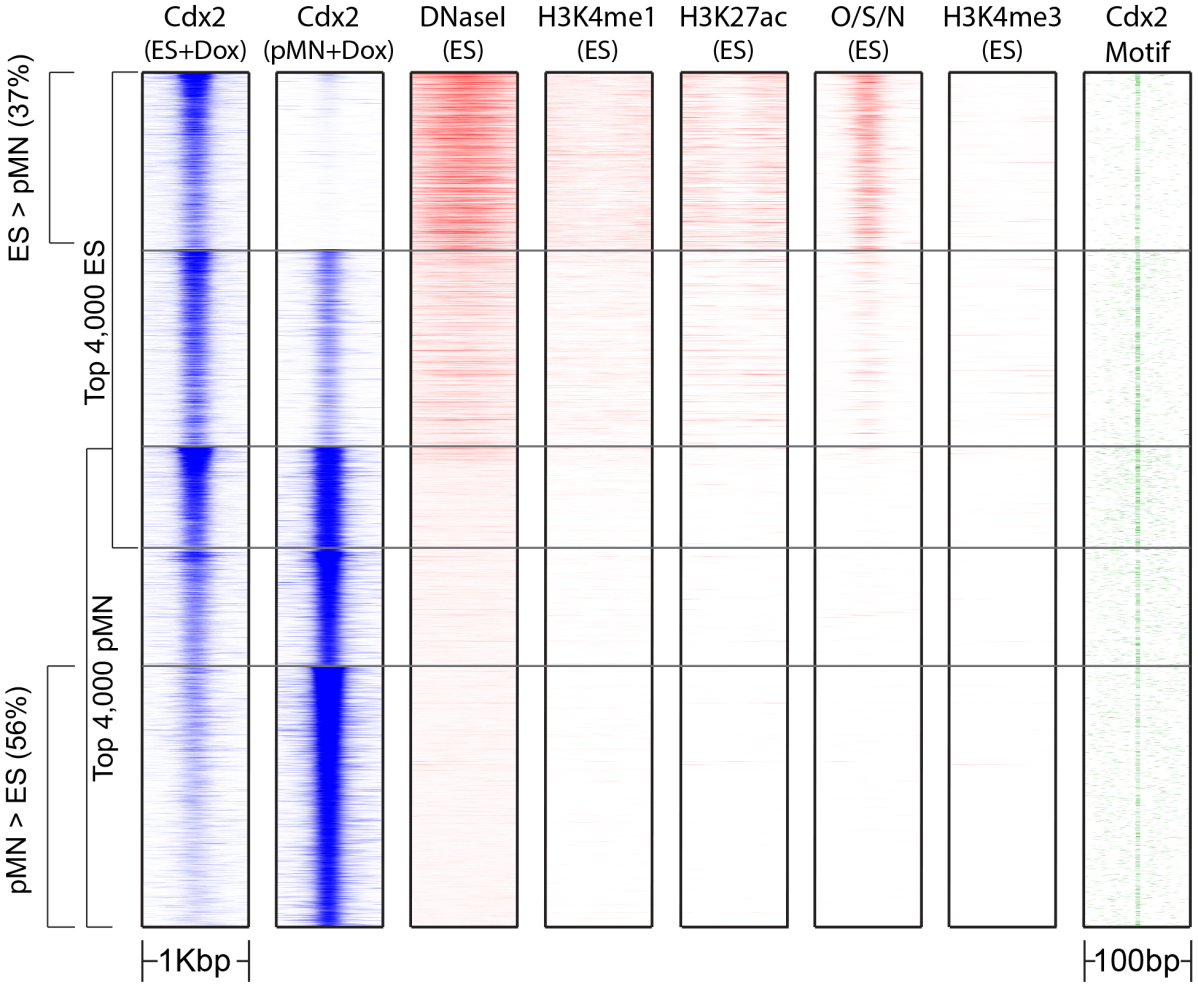
Clustergrams of the top 4,000 binding events in ES+Dox and pMN+Dox conditions, clustered according to MultiGPS differential binding calls. Cdx2 binding is compared with ES cellular state information, including DNaseI-seq, chromatin marks, and Oct4/Sox2/Nanog TF ChIP-seq (O/S/N). Similar results comparing ES+Dox and endoderm+Dox conditions are presented in Figure S3.

Since MultiGPS identifies a large proportion of condition-specific Cdx2 binding events without finding any evidence for a corresponding change in Cdx2's DNA-binding preference, we asked whether ES cell genomic context could predict the observed condition-specific binding of Cdx2 after induction. To answer this question, we examined the ES genomic patterns at the locations of Cdx2 sites that are significantly enriched in ES+Dox cells according to MultiGPS. Interestingly, we found that ES+Dox-specific Cdx2 sites are enriched for ES signatures of chromatin accessibility (DNaseI hypersensitivity), enhancers (H3K4me1 and H3K27ac ChIP-seq), and TF binding (Oct4, Sox2, and Nanog ChIP-seq), but not active transcription (H3K4me3 ChIP-seq) (Figure 5). Conversely, pMN+Dox-specific Cdx2 sites and endoderm+Dox-specific Cdx2 sites show no enrichment for these ES cell chromatin signatures (Figure 5 & Figure S3).

**Figure 6:**
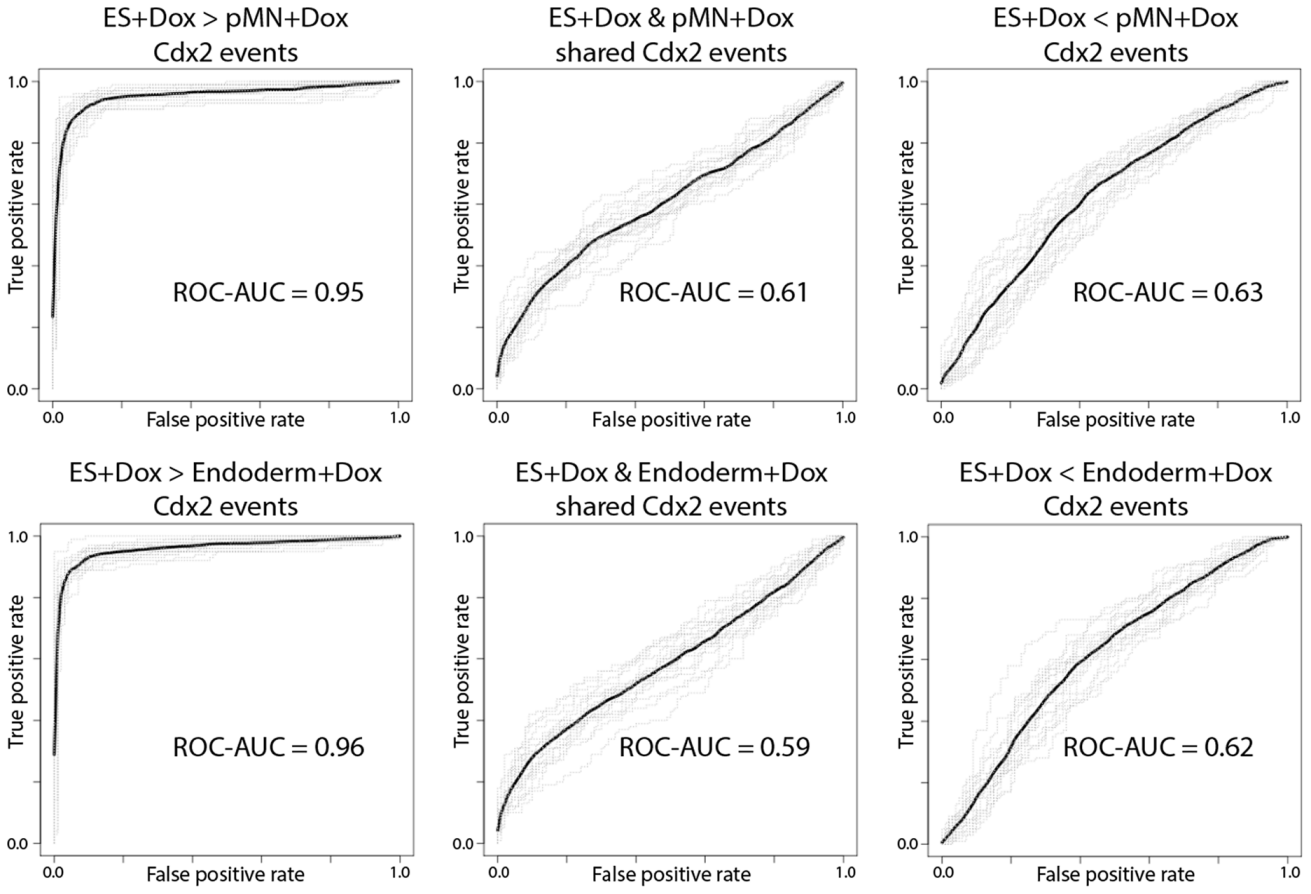
Predictive performance of SVMs trained using ES chromatin state information when discriminating between subsets of Cdx2 binding events and unbound Cdx2 motif instances.

To more rigorously test the capacity of ES cell genomic context to predict ES+Dox-specific Cdx2 binding events, we trained support vector machines (SVMs) to classify Cdx2 binding events vs. unbound Cdx2 motif instances using the read count information from a collection of 55 ES experiments (2 DNaseI-seq, 13 histone modification ChIP-seq, 35 TF, co-activator and chromatin modifier ChIP-seq, and 5 Pol2 ChIP-seq experiments). Cross-validation was used to generate disjoint training and test sets (see Methods). Our SVMs discriminate ES+Dox-specific Cdx2 sites from unbound sites with an area under true-positive vs. false-positive curve (AUC) of 0.95–0.96, suggesting that the pre-existing genomic context in ES cells is highly predictive of future Cdx2 binding. Conversely, our SVMs are unable to discriminate pMN+Dox-specific Cdx2 sites from unbound Cdx2 motif instances using ES genomic context (AUC = 0.63, Figure 6). Our results therefore suggest that condition-specific Cdx2 binding events are more likely to be located in genomic regions that already displayed regulatory activity or accessibility before Cdx2 expression was induced.

### Condition-independent Cdx2 binding is associated with higher cognate motif affinity

Since condition-specific Cdx2 binding events appear highly correlated with immediately pre-existing genomic context, we reasoned that the condition-independent Cdx2 sites that are bound in multiple conditions might also display the same associations. For example, Cdx2 sites that are bound in two conditions may represent locations that happened to have pre-existing regulatory activity or accessibility in both conditions. Surprisingly, the Cdx2 sites bound in both ES+Dox and pMN+Dox conditions are not enriched for accessibility, enhancer chromatin marks, or TF binding in ES cells (Figure 5). Furthermore, SVMs trained as before are unable to discriminate between these shared Cdx2 sites and unbound motif instances using ES genomic context information (AUC = 0.61, Figure 6). These results suggest that the condition-independent Cdx2 sites are not determined by pre-existing genomic context, in contrast with the condition-specific sites.

**Figure 7:**
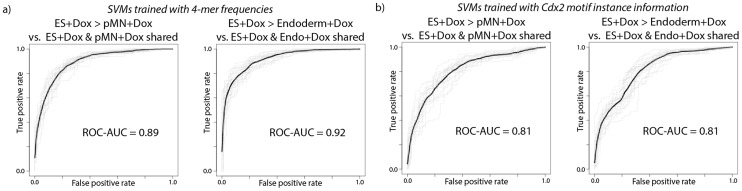
Predictive performance of SVMs when discriminating between condition-specific and condition-independent subsets of Cdx2 binding events, where SVMs are trained using a) all 4-mer frequencies or b) Cdx2 motif information in 200 bp windows around the binding events.

Given that the condition-independent Cdx2 sites do not seem to have any distinguishing chromatin features before Cdx2 induction, we asked how Cdx2 recognizes these sites regardless of genomic context. We hypothesized that such sites may have sequence features that enable condition-independent binding. To test this hypothesis, we trained SVMs to discriminate condition-independent Cdx2 sites from condition-specific Cdx2 sites using only 4-mer word frequencies in 200 bp windows around the sites. Surprisingly, even these crude sequence features were sufficient to discriminate between the two types of sites (AUC = 0.89–0.92, Figure 7a), suggesting that some sites contain sequence information that enables condition-independent Cdx2 binding. We next used the discriminative motif finders DEME and DECOD [24], [25] to determine which sequence motifs discriminate between Cdx2 site types. Interestingly, both tools returned the primary Cdx2 motif as being the most discriminative, even though most condition-specific and condition-independent sites contain instances of the same primary motif. This apparent contradiction is resolved by considering features of the motif instances in each set of Cdx2 sites. SVMs trained with just three simple primary Cdx2 motif-related metrics – the maximum motif score in the 200 bp window around sites, the number of motif instances above a threshold, and a score that integrates motif scores across the entire 200 bp window [26] – were able to discriminate between condition-independent and condition-specific sites with reasonable accuracy (AUC = 0.81, Figure 7b). In other words, the strength and multiplicity of motif instances are somewhat predictive of condition-independent Cdx2 binding.

Taken together, our results suggest that sequence information allows Cdx2 to act as a pioneer TF at some sites, overriding the lack of pre-existing accessibility or chromatin markers.

## Discussion

MultiGPS provides a principled platform for the analysis of differential protein-DNA binding across multiple experimental conditions by preferring consistent binding locations across related experiments while also modeling condition-specific experimental parameters. Rather than treating reads from all experiments as equivalent, MultiGPS models experiment-specific read distributions around binding events. MultiGPS can thus correctly analyze collections of related ChIP experiments that were performed according to different protocols such as mixtures of related ChIP-seq and ChIP-exo [27] experiments. As demonstrated above, MultiGPS improves the quantification of ChIP enrichment at binding events in comparison with the typically used window-counting approaches, thus enabling more sensitive analyses of differential binding enrichment between conditions.

Since MultiGPS prefers but does not force binding events to align across experiments, it may also be used to study possible forms of differential binding activity that we did not illustrate. For instance, it may be of interest to examine locations where the underlying read evidence overrides the MultiGPS inter-experiment prior, resulting in differing reported binding locations across experiments. Such locations may represent shifts in binding location between conditions, which may be useful for studies of nucleosome positioning or regulators that might bind alternate nearby locations in different conditions.

We demonstrated that MultiGPS can characterize condition-specific binding and then used MultiGPS to characterize the nature of both condition-specific and condition-independent binding of Cdx2. Our results suggest that many condition-specific Cdx2 binding events are located in regions that had pre-existing regulatory activity, thus agreeing with hypotheses proposed to explain the observed binding of other developmental TFs [1]–[3]. However, Cdx2 also appears to act as a ‘pioneer’ at a subset of sites that are bound condition-independently. Our analysis suggests that such sites on average contain stronger and more frequent Cdx2 motif instances than condition-specific sites, thus suggesting a possible mechanism by which condition-independent sites can be bound regardless of preexisting genomic context. These findings also accord with our recent demonstration that TF combinations can override pre-existing cellular state to synergistically bind composite motifs during motor neuron programming [28], perhaps pointing to a deeper relationship between sequence information and ‘pioneer’ binding activity.

## Methods

### The MultiGPS mixture model

In our previously described GPS [16] and GEM [17] approaches to binding event detection, ChIP sequencing data are modeled as being generated by a mixture of binding events along the genome, and an Expectation Maximization (EM) learning scheme is used to probabilistically assign sequencing reads to binding event locations. The assignment of reads is achieved via an empirically estimated multinomial distribution, Pr(*r_n_*|*x*), which gives the probability of observing read *r_n_* from a binding event located at genomic coordinate *x*. Conceptually, every base position is treated as a potential binding event, although the use of a sparse prior [29] has the effect of allowing only a small subset of these potential binding events to take responsibility for observed reads and survive the EM training process.

In MultiGPS, we decouple the relationship between a binding event's index and its spatial (genomic) location. Specifically, we introduce a vector of component locations ***μ*** where *μ_j_* is the genomic location of event *j*. We initialize a large number of potential events, *M*, such that the events are evenly spaced in 30 bp intervals along the genome. Note, however, that the use of a sparse prior will again result in only a subset of events remaining active in the model after training (i.e. components having mixing probability *π_j_*>0; see MAP estimation of ***π*** below). In the new mixture model, the likelihood of observing the *N* total ChIP read locations ***r*** is given by:
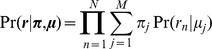
where Pr(*r_n_*|*μ_j_*) is the distribution over ChIP-seq read positions conditioned on membership in a binding event at location *μ_j_*. This distribution is initialized to a strand-specific shape typical of many ChIP-seq datasets (see Figure S5), and is iteratively re-estimated during EM training using the distribution of reads observed around high-confidence binding site locations. The above expression calculates the observed data likelihood of a mixture model by taking the product over all reads, where each read averages over each possible binding event that may have caused it. This extension of the model allows us to apply prior knowledge directly to the positions of the binding events (***μ***), without affecting the binding event strength estimation or the sparsity-promoting prior, which continues to act on raw expected read counts.

We introduce a Bernoulli prior over each genomic location where each element *k_i_* of the parameter ***k*** corresponds to the probability that location *i* is a binding event (that is, *i* 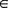 ***μ***). This prior assumes that there can be only one or zero binding events at a single position and that binding positions are selected independently along the genome according to this weighting. The prior assigns likelihoods to a set of binding events on a genome of size *L* as follows:
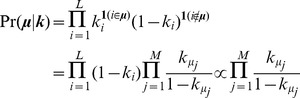



As in the original framework, the latent assignments of reads to binding events are represented by the vector ***z***. The complete-data log posterior can thus be derived as follows:
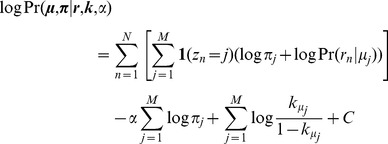
Here, *C* is a normalization constant that does not involve any of the terms to be optimized. It can be seen that the overall binding event sparsity-inducing negative Dirichlet prior *α* acts only on the mixing probabilities ***π***, which controls the total fraction of reads assigned to each binding event, and the positional prior ***k*** acts only on the binding event locations ***μ***. Therefore, the E-step that calculates the relative responsibility of each binding event in generating each read is unchanged from our original framework, following standard mixture model approaches:
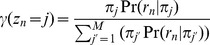
Furthermore, the maximum a posteriori probability (MAP) estimation of ***π*** is also unchanged:
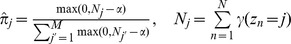
where *N_j_* is the effective number of reads assigned to binding event *j*. The *α* parameter can thus be interpreted as the minimum number of ChIP-seq reads required to support a binding event remaining active in the mixture model. We set the value of *α* per experiment to be the maximum number of reads that would be expected to occur (*p*>10^−7^) in a window equal to the effective range of the binding distribution should the experiment's reads be distributed uniformly along the mappable portion of the genome.

We can estimate ***μ*** component-wise since it only participates in sums in the log likelihood. However, no closed form solution exists since the prior ***k*** has no parametric form. We can determine the MAP (integer) value of *μ_j_* by simply enumerating over all possible values of *μ_j_*. Specifically, the MAP value of *μ_j_* is: 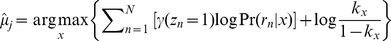. If the maximization step results in two components sharing the same location, they are combined in the next iteration of the algorithm.

One practical use for the positional prior ***k*** is to bias the estimated binding locations towards biologically appropriate base positions. For example, a TF's position weight matrix scores along the genome can be directly encapsulated in ***k*** in the above framework. As described previously for our GEM approach [17], we can estimate binding motifs from current estimates of binding locations, and reciprocally use those motifs as prior information to re-estimate binding event locations. Note that motif priors are incorporated quite differently in GEM and MultiGPS. In practice, MultiGPS uses MEME [30] to discover a set of over-represented motifs in the top 500 most enriched binding events (80 bp windows), chooses the motif with the highest true-positive vs. false-positive AUC for discriminating bound regions from random sequences (if any motif AUC≥0.7), and incorporates the genomic log-odds scores for that motif in the positional prior.

Unlike our previously described approaches, MultiGPS incorporates an additional mixture component that explicitly models noise (i.e. reads arising from nonspecific binding and independent of any binding event). Whereas binding component read distributions have approximately finite support (and therefore only allow binding events to take responsibility for reads in their local vicinity), the noise component is defined as having a global distribution. The form of the noise distribution can be defined as uniform or can be parameterized using the read density observed in a control experiment. In the latter case, the shape of the noise distribution is defined by smoothing the control experiment's read counts using a 50 bp sliding window (adding fractional pseudocounts to 50 bp windows that contain no control reads).

For a more efficient and stable training process, some parameters in MultiGPS are re-estimated only periodically, including the form of the binding event read distribution, the noise component mixing probability (*π_M_*_+1_), and the binding motif position weight matrix. We can therefore think of MultiGPS as an instance of a generalized EM algorithm. Generalized EM algorithms increase the expected log likelihood in each M step without necessarily achieving a maximum in each iteration (as in the original EM algorithm) [31]. Convergence to a local optimum is guaranteed with generalized EM algorithms, as it is with the EM algorithm [31].

As with GPS and GEM, MultiGPS filters predicted binding events to require that their associated read counts are significantly enriched (*p*<10^−3^, Benjamini-Hochberg corrected Binomial test) over the corresponding read count from an appropriately normalized control experiment, such as a mock-IP experiment. The control experiment normalization factors are estimated via regression on the read count ratios in 10 Kbp windows. Control read counts are associated with individual binding events via maximum likelihood assignments using the trained model (i.e. assigning control reads to binding events without changing the ***π*** and ***μ*** parameters learned from the ChIP data).

### Analyzing multiple experimental conditions in MultiGPS

MultiGPS can be run in a multi-condition analysis mode by providing multiple input datasets and structured annotation as to how these datasets are related (i.e. which datasets represent technical or biological replicates of others, which collections of datasets represent distinct experimental conditions, and which datasets serve as controls for others). MultiGPS then runs semi-independent mixture model training across all provided data. Since reads from distinct conditions are not pooled, MultiGPS can maintain condition-specific and replicate-specific parameters, including distinct binding event read distributions per replicate, distinct noise component read distributions and mixing probabilities per replicate, and distinct binding motifs per condition. However, the goal is to report binding event locations that are consistent across conditions. This is achieved using another form of prior information during the maximization of binding event locations ***μ***.

**Figure 8:**
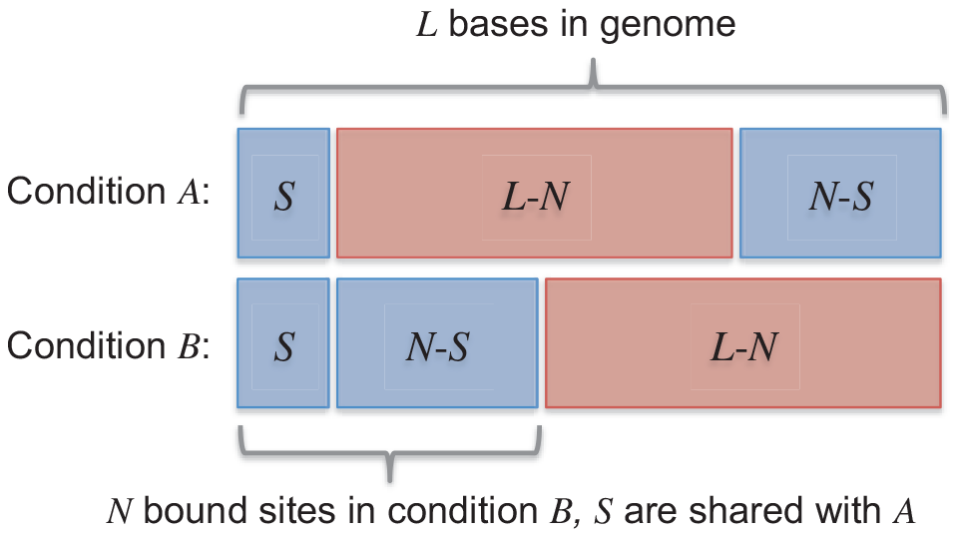
Schematic of bound and unbound bases in two conditions.

We motivate our approach by imagining a TF that binds to *N* locations in cellular condition *A* and *N* locations in cellular condition *B*. In typical analysis scenarios, the number of bound locations will be much fewer than the number of bases on the genome (i.e. 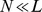), and a non-zero set of *S* locations will be bound in both *A* and *B* conditions. We present the model for two conditions with a symmetric number of binding sites here for notational simplicity, but note that the same process can be applied to any number of conditions with more complex binding site sharing patterns. A schematic example (not to scale) of bound and unbound bases in two conditions as a fraction of the genome is shown in Figure 8.

Now, the distribution that generates binding positions is extended from the single-condition case of a Bernoulli distribution to a multivariate Bernoulli distribution. As suggested by the schematic in Figure 8, this distribution generates a sample from {(0,0),(0,1),(1,0),(1,1)} at each base in the genome, where each element in a sample corresponds to whether a binding site is present at that position in that condition. This generative model induces the following distribution over genome positions *i* with respect to binding site positions in conditions *A* and *B*:
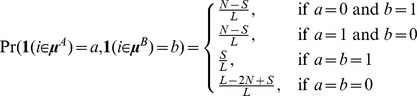
We parameterize the above distribution during each iteration of the MultiGPS algorithm by choosing appropriate values for *N* and *S* (*L* is fixed, being the length of the genome). While *N* can be taken from MultiGPS' current estimate of the number of binding events in each condition, we do not typically know *S*. We therefore define *S* by setting the ratio *S/N* as described below.

We need to know the contribution of the location prior 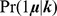 in the optimization step for the binding site locations ***μ***. For the multi-condition analysis, we jointly optimize two binding sites when they fall within 100 bp of each other (range chosen empirically as the maximum range for which the inter-experiment prior will have an effect at most binding events, see Figure S7). The model optimization step determines whether the two binding positions in question are separate (and therefore two site-specific positions contribute to 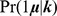) or shared (and therefore one shared site contributes to 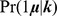). All other bases will be the same during this optimization since all other binding sites are fixed, and can be ignored in this step. Using the distribution above gives the following contribution to the prior 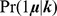:
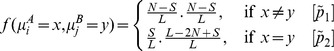
where 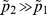 in most experimental studies. If the binding events share a location across conditions, we choose the optimal shared position *w* by maximizing the expected complete-data log posterior (with terms not affecting the minimization omitted) as follows:
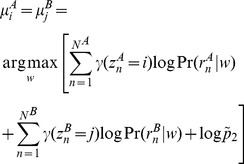
Alternatively, the two binding component locations are independent, in which case the two positions are optimized independently:
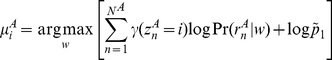


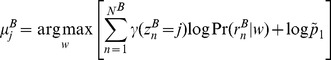



The decision to use the coupled or uncoupled estimate is based on which scenario yields the higher expected complete-data log posterior probability. Higher values of the ratio *S/N* encourage the coupling of nearby binding event locations across conditions by increasing 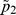 with respect to 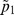 (see Figures S6 & S7). In MultiGPS, we set the ratio *S/N* to be equal to 0.9, although in practice we observe few differences in the proportion of aligned binding components when varying the ratio in the range 0.5<*S/N*<0.99. This is because a number of nearby genomic locations give similar probabilities when maximizing *μ_j_* (Figure S7), allowing the penalties associated with moving the components away from the optimal positions in each condition to be overridden by the positive-valued 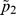 prior over a range of *S/N* ratios. Note, however, that MultiGPS will still prefer the uncoupled scenario in situations where the read evidence supports distinct binding locations across conditions. This behavior represents a data-driven joint analysis mode that weighs the statistical confidence given by the reads against prior knowledge of the experimental setup in a probabilistically optimal way. We also note that positional prior terms encapsulating per-condition TF position weight matrix scores can be accounted for in the ***μ*** maximization terms above in a manner analogous to that described in the previous section. MultiGPS can therefore account for both motif positional priors and the inter-experiment prior.

Assessing all possible scenarios of coupled and uncoupled binding events during the update of each *μ_j_* becomes prohibitive when analyzing more than two conditions. Therefore MultiGPS assesses a limited number of scenarios when updating *μ_j_* in such cases: 1) event *j* is uncoupled across all conditions; 2) event *j* is coupled with a corresponding event in one other condition; or 3) event *j* is coupled with corresponding events in all other conditions. The scenario that yields the best overall likelihood is chosen.

### Detecting differential binding

A table containing the replicate-specific read counts associated with each binding event is generated from the MAP-estimated responsibilities ***γ***. MultiGPS uses the edgeR Bioconductor package [6] to detect differential ChIP enrichment between conditions from the read count table. We use edgeR's TMM method to calculate normalization factors, and the glmLRT method to calculate likelihood ratios. In the Cdx2 example described here, we used a fixed overdispersion parameter of 0.15 across all experiments, which results in a stricter definition of significant differential enrichment than the overdispersion parameters estimated by edgeR.

### Benchmarking analysis on simulated ChIP-seq data

To computationally simulate multi-condition ChIP-seq data, we defined a hypothetical system in which a protein has 20,000 binding events in the mouse genome (version mm9). The relative strengths of each of these binding events was drawn randomly from a distribution of relative read counts observed for Cdx2 binding events in our pMN ChIP-seq experiments. For two hypothetical experimental conditions, A & B, we randomly chose 20% of the binding events to be differentially enriched in condition A with respect to B, and we modify the relative binding event strengths of these sites such that they are 4-fold (or 8-fold in separate simulations) greater in condition A versus B. We similarly chose a non-overlapping 20% of binding events to be differentially enriched in condition B with respect to A. The binding events were placed along the genome in 10 Kbp intervals.

We then generated 20 million read positions for each of two replicates in each of the two conditions. To reflect the typical signal-to-noise ratio observed in real ChIP-seq experiments, 95% of the read positions are spread randomly across the entire genome. The remaining reads (averaging 1 million per replicate) are distributed amongst the binding events according to the relative strength of the event in each relevant condition, and accounting for read sampling noise using a negative binomial distribution with an over-dispersion parameter of 0.1. The MA plot in Figure 2a shows the log_2_ mean read count and log_2_ fold difference for each binding event in the simulated experiments. The position of generated reads with respect to the defined binding event location is drawn from a bimodal distribution typical of ChIP-seq binding sites (Figure S5).

We ran the following binding event analysis methods on the simulated data: a) MultiGPS on the entire dataset, using default parameters with the exception of turning off the use of sequence information and the motif prior (since motif information was not used in generating the simulated data); b) MultiGPS without the inter-experiment prior or the motif prior on the entire dataset, which has the effect of calling binding events in each condition independently; c) MACS [18] using default parameters on each condition independently, merging reads across replicates; and d) SISSRs [19] using default parameters (with the exception of using a p-value cutoff of 0.05) on each condition independently, merging reads across replicates. For binding event list comparison approaches, per-condition events were compared with each other using a 200 bp window. In other words, if an event prediction in one condition is located within 200 bp of an event prediction in the other condition, it is treated as being in the intersection of the binding event list comparison, and thus not differentially bound. EdgeR [6] was run either internally in MultiGPS (as described above) or, using the same parameters, on read count tables built by counting reads that overlap the peak regions found by MACS or SISSRs. We also ran DBChIP [9] using default parameters with the exception of an FDR threshold <0.01 and using the MACS peaks as inputs. Sensitivity and specificity in Figures 2 and S1 are defined by comparing predicted binding events to the positions of the simulated differential binding events using a 100 bp window.

### SVM analysis

Support vector machines were trained using the libSVM [32] interface in Bioconductor (e1071). In all cases, classification accuracy was determined using a randomly selected held-out test set of 100 datapoints, and training of each SVM application was repeated 20 times (using different held-out test sets each time) to calculate average true-positive vs. false-positive AUC values.

To train SVMs using ES chromatin state data, we first gathered 55 mouse ES ChIP-seq and DNaseI-seq experiments from a variety of sources [33]–[42]. We defined positive training sets from the top-most Cdx2 binding events for each condition-specific and condition-independent permutation (up to a maximum of 4,000 binding events), and we also defined a negative training set of 10,000 matches to the Cdx2 cognate binding motif (as defined by UniProbe [43]) that were not bound by Cdx2 in any experiment. Reads were counted in 1,000 bp windows around each of the positive and negative locations for each of the 55 mouse ES experiments, and SVMs were trained on the resulting 55-dimensional vectors without any normalization.

SVMs were trained on *k*-mer frequencies by enumerating the occurrences of each 4-mer (accounting for reverse-complement redundancies) in 200 bp windows around each of the top-most Cdx2 binding events for each condition-specific and condition-independent permutation (up to a maximum of 4,000 binding events). Similarly, SVMs were also trained on three pieces of information from the same 200 bp windows: the maximum log-likelihood ratio score for the Cdx2 motif in the window; the number of matches to the motif in the window that score more than a 5% FDR threshold; and the probability of binding occupancy in the window [26].

### ChIP experiments & processing

An ES cell line harboring Dox-inducible Flag-tagged Cdx2 was generated as previously described [44]. Anti-Flag ChIP-seq experiments were performed as previously described [44] after 24 hours of Dox-induced expression of Cdx2 in the ES cells or in motor neuron progenitors or endoderm cells that were differentiated from the same ES cell line. Differentiation of the ES cells to pMN and endoderm lineages was also described previously [20], [21]. Mock IP control experiments were performed using the same system. Sequenced ChIP-seq reads were mapped to the mm9 reference genome using Bowtie [45]. ChIP-seq data generated during this study were deposited in GEO under accession numbers GSE39433 and GSE39435.

### Availability

MultiGPS is available as an open-source Java package, released under the MIT license, from: http://mahonylab.org/software and https://github.com/shaunmahony/seqcode. Simulated multiple condition ChIP-seq datasets are also available from the same webpage.

A summary of this paper appears in the proceedings of the RECOMB 2014 conference, April 2–5 [46].

## Supporting Information

Figure S1 **a)** MA plot displaying the mean read count and log fold ratio distributions of the simulated ChIP-seq dataset in which 40% of binding events are defined as 8-fold differentially enriched in one condition versus the other. Defined differential events highlighted in blue, non-differential in gray. **b)** Sensitivity and **c)** specificity of various approaches when predicting differentially bound events. Results are broken out by quintile on the mean read count across conditions (i.e. based on x-axis in **a)**).
(TIF)

Figure S2 Detected binding event counts (**a**,**c**) and average distance from binding event prediction to defined binding position (**b**,**d**) for various methods when predicting events in simulated ChIP-seq datasets. Results are presented broken down by quintile of the mean absolute read count associated with the binding event across conditions.
(TIF)

Figure S3 Clustergrams of the top 4,000 binding events in ES+Dox and endoderm+Dox conditions, clustered according to MultiGPS differential binding calls. Cdx2 binding is compared with ES chromatin state information, including DNaseI-seq, chromatin marks, and Oct4/Sox2/Nanog TF ChIP-seq (O/S/N). Similar results comparing ES+Dox and pMN+Dox conditions are presented in Fig. 4.
(TIF)

Figure S4 Predictive performance of SVMs trained using Cdx2 motif information when discriminating between condition-specific and condition-independent subsets of Cdx2 binding events.
(TIF)

Figure S5 Initial strand-specific distribution Pr(*r_n_*|*μ_i_*) used in the multiGPS mixture model.
(TIF)

Figure S6Log prior differences (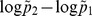) as a function of varying *S*/*N*.(TIF)

Figure S7 Log-likelihood assigned by MultiGPS to various positions around the optimal binding location, as a function of the number of reads associated with the binding event. Events with higher read counts have more sharply peaked log-likelihood landscapes, since there is more evidence pointing towards the optimal binding location. For illustration, we placed gray shaded bars representing a log-likelihood range of 10 around the peak of each log-likelihood distribution. The shaded bars illustrate the degree to which a typical cross-condition prior value (see Figure S6) can affect the binding location update step. If binding events are detected in nearby locations in each condition, the cross-condition prior will encourage them to align by overriding the optimal log-likelihood value found from read evidence alone. However, if events are associated with high read counts, the window in which the cross-condition prior can have an effect is reduced. This allows MultiGPS to detect truly distinct, but nearby located, condition-specific binding events if sufficient read evidence exists to support their existence in the model.
(TIF)

Table S1 MultiGPS increases sensitivity for detecting cell-type specific binding events. edgeR was used to do a three-condition analysis across the Tier 1 cell types for each protein, where each cell type/protein pair was performed twice. Recommended edgeR practices were used to estimate normalization factors and overdispersion amounts, and sites that had any condition-specific signature (testing for any of the coefficients in the regression model to be nonzero) were identified. The table reports the number of sites reported as significant by edgeR (FDR<0.01).
(DOCX)
