# Supplementary figures and images for: An Integrated Model of Multiple-Condition ChIP-Seq Data Reveals Predeterminants of Cdx2 Binding
Source: PLoS Comput Biol. 2014 Mar 27;10(3):e1003501. doi: 10.1371/journal.pcbi.1003501 (PMC3967921; doi:10.1371/journal.pcbi.1003501)

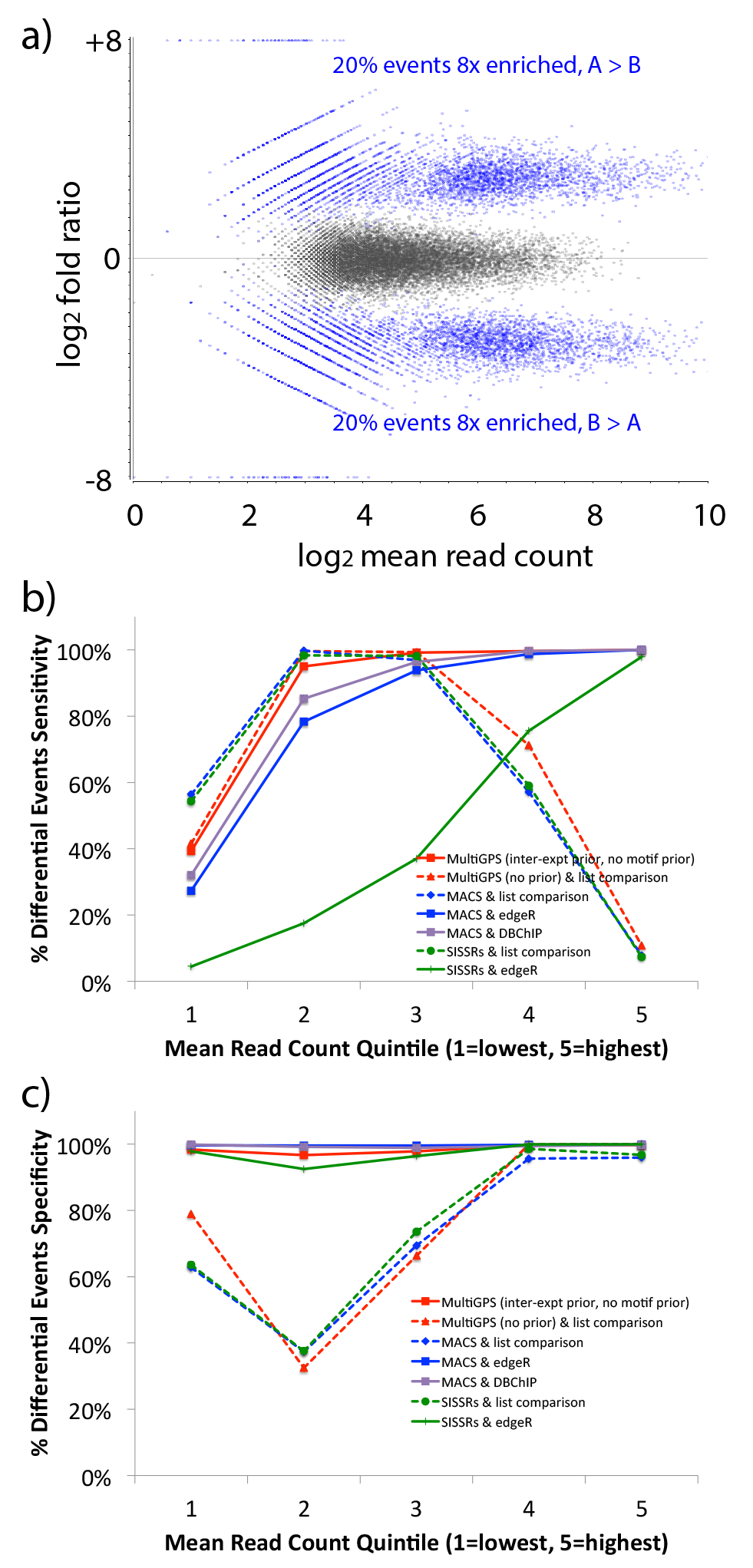

Supplement: Figure S1 — a) MA plot displaying the mean read count and log fold ratio distributions of the simulated ChIP-seq dataset in which 40% of binding events are defined as 8-fold differentially enriched in one condition versus the other. Defined differential events highlighted in blue, non-differential in gray. b) Sensitivity and c) specificity of various approaches when predicting differentially bound events. Results are broken out by quintile on the mean read count across conditions (i.e. based on x-axis in a)). (TIF) [file pcbi.1003501.s001.tif]

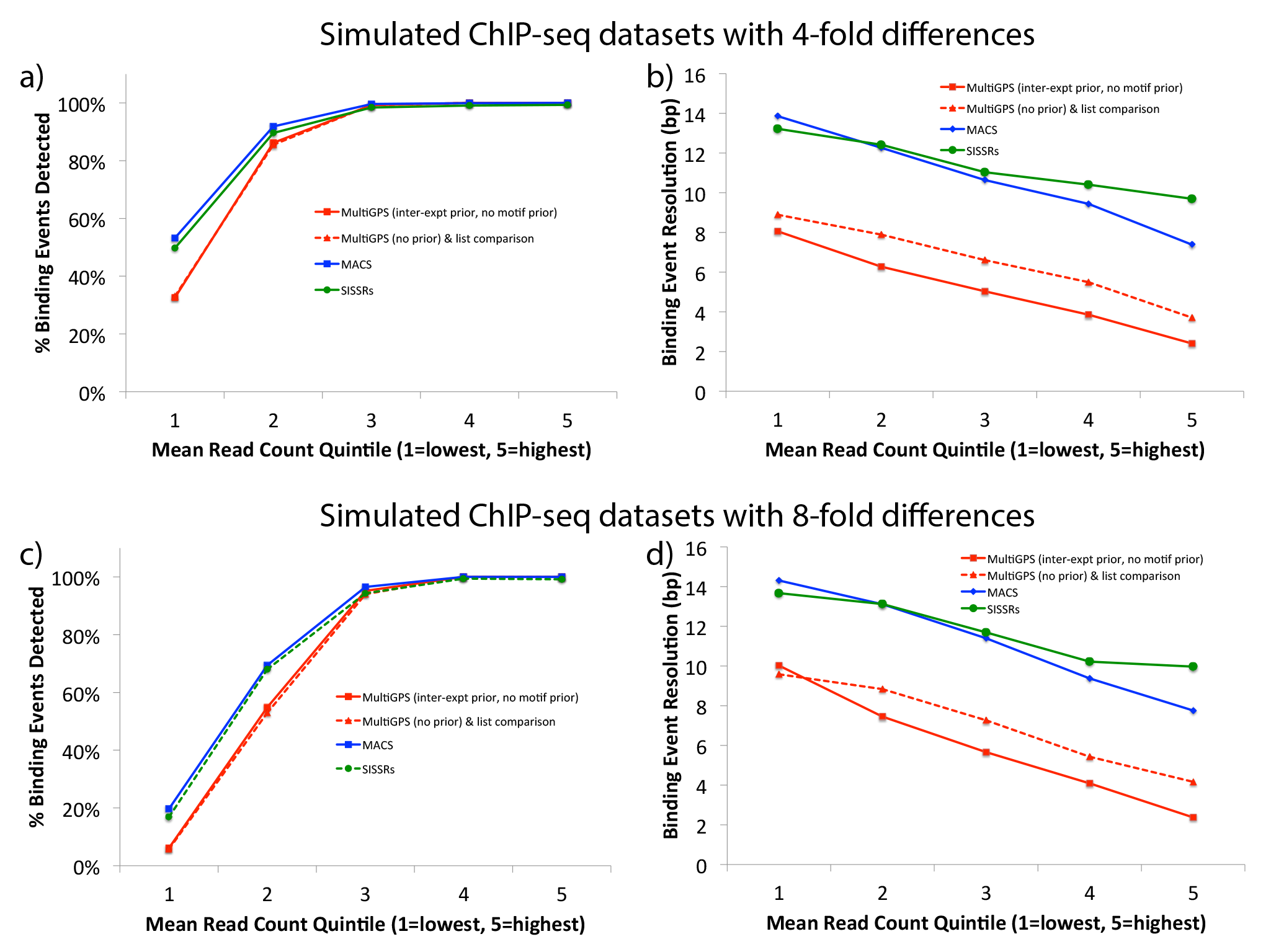

Supplement: Figure S2 — Detected binding event counts (a,c) and average distance from binding event prediction to defined binding position (b,d) for various methods when predicting events in simulated ChIP-seq datasets. Results are presented broken down by quintile of the mean absolute read count associated with the binding event across conditions. (TIF) [file pcbi.1003501.s002.tif]

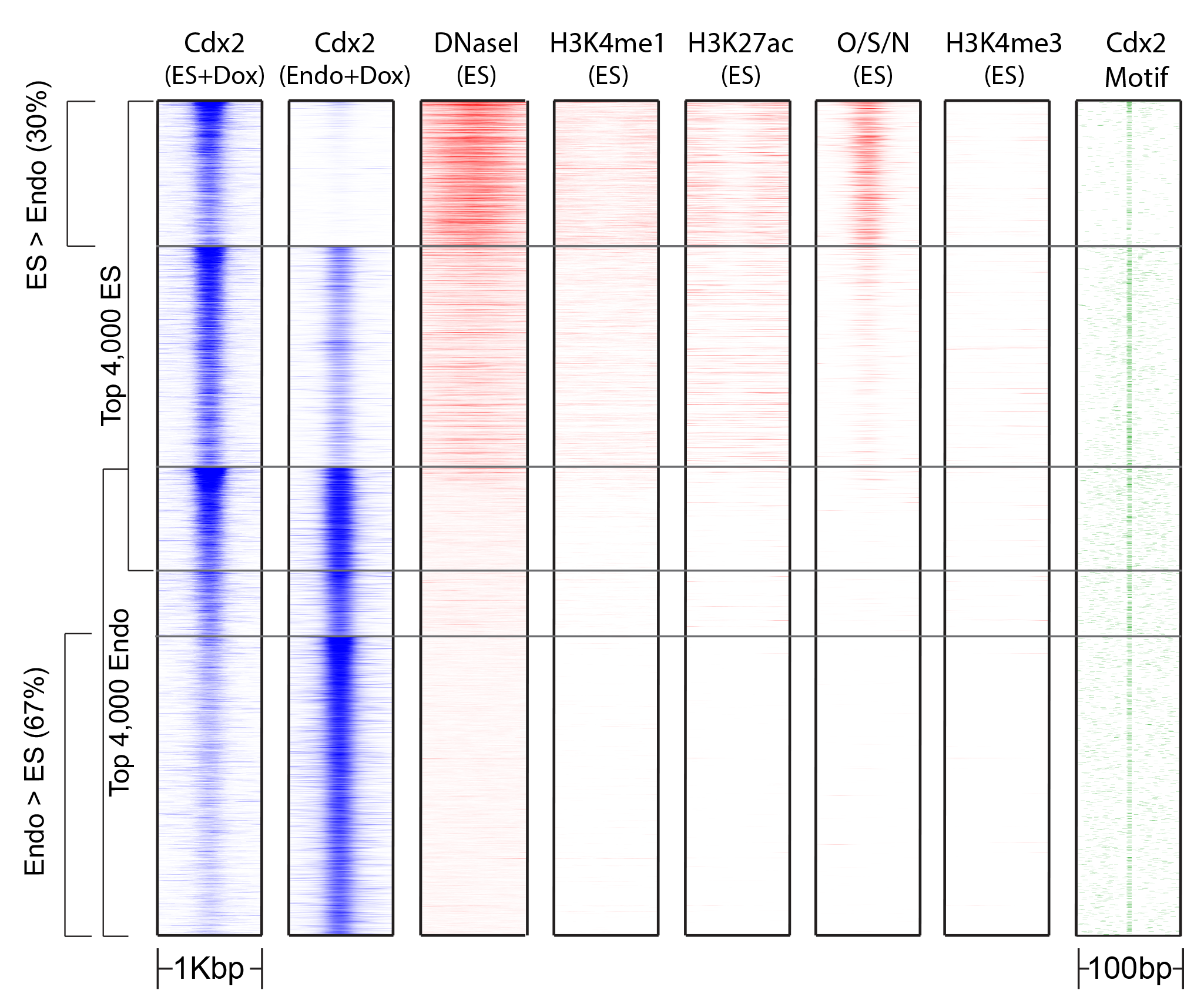

Supplement: Figure S3 — Clustergrams of the top 4,000 binding events in ES+Dox and endoderm+Dox conditions, clustered according to MultiGPS differential binding calls. Cdx2 binding is compared with ES chromatin state information, including DNaseI-seq, chromatin marks, and Oct4/Sox2/Nanog TF ChIP-seq (O/S/N). Similar results comparing ES+Dox and pMN+Dox conditions are presented in Fig. 4. (TIF) [file pcbi.1003501.s003.tif]

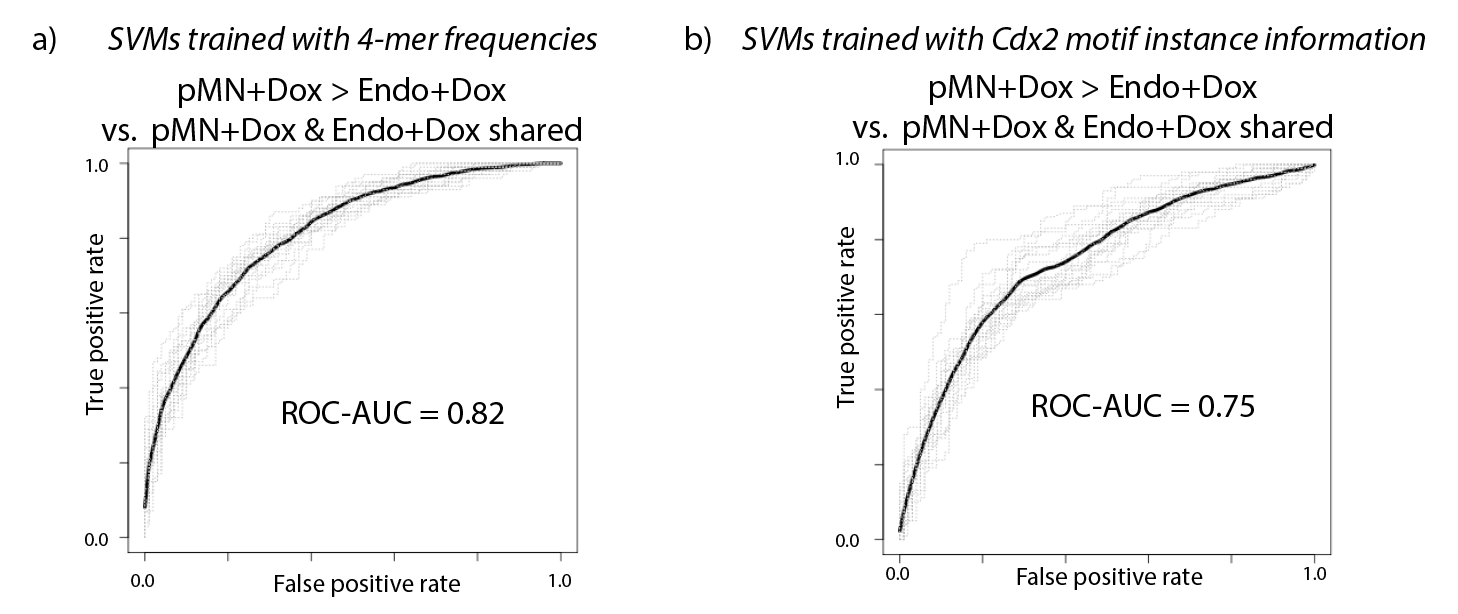

Supplement: Figure S4 — Predictive performance of SVMs trained using Cdx2 motif information when discriminating between condition-specific and condition-independent subsets of Cdx2 binding events. (TIF) [file pcbi.1003501.s004.tif]

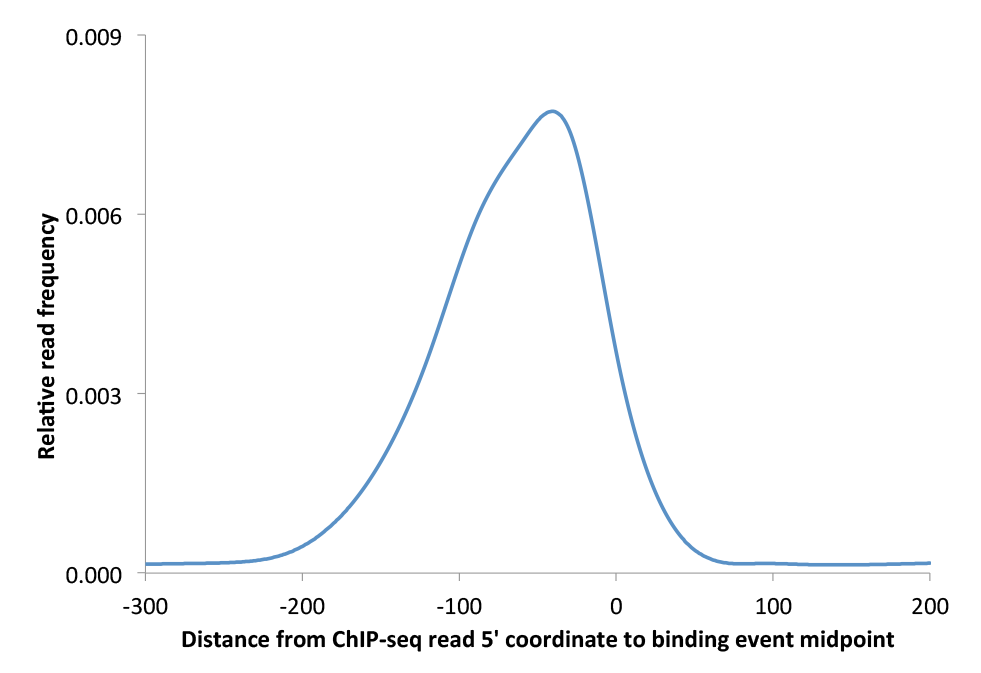

Supplement: Figure S5 — Initial strand-specific distribution Pr(rn|μi) used in the multiGPS mixture model. (TIF) [file pcbi.1003501.s005.tif]

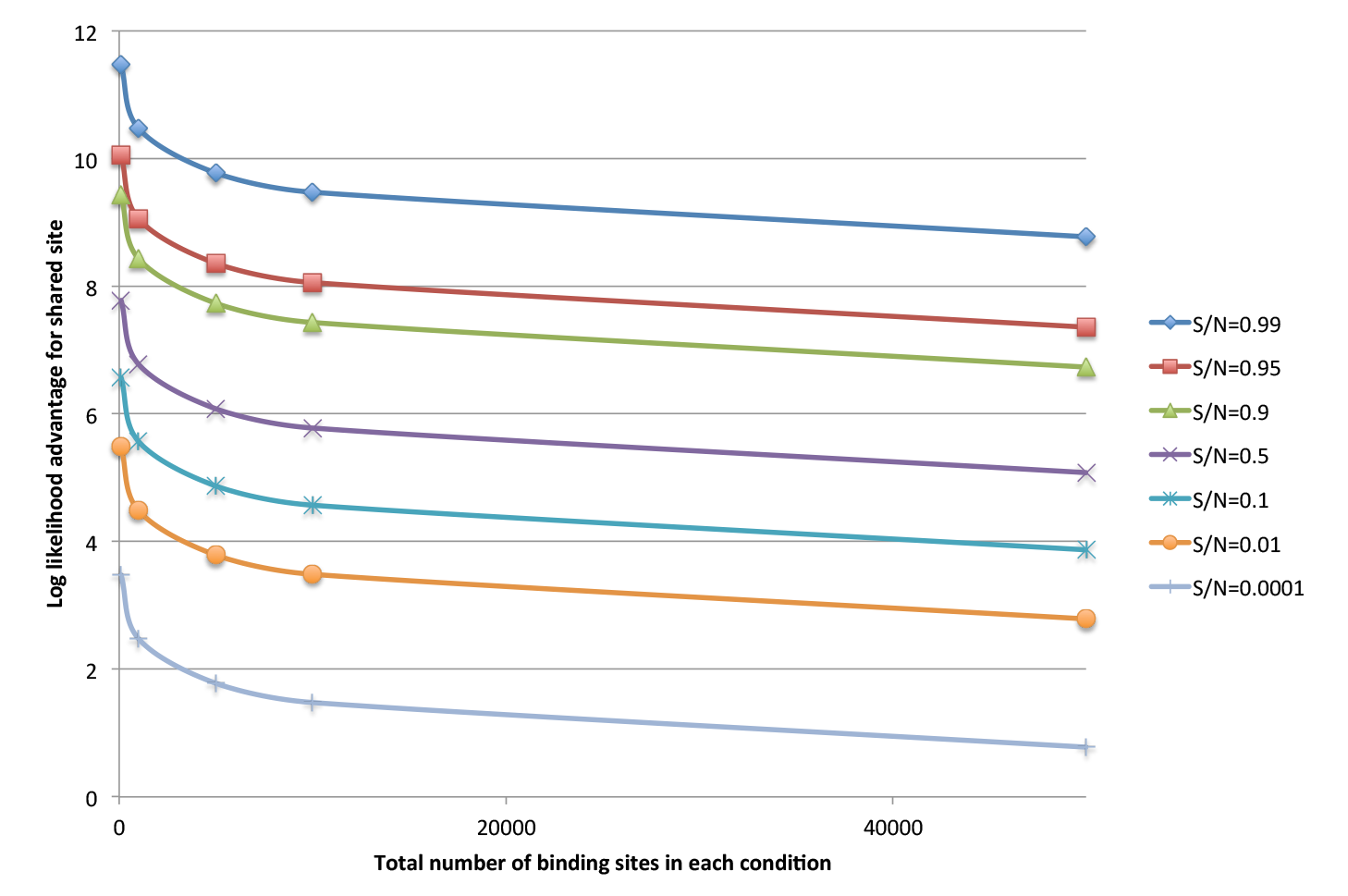

Supplement: Figure S6 — Log prior differences () as a function of varying S/N. (TIF) [file pcbi.1003501.s006.tif]

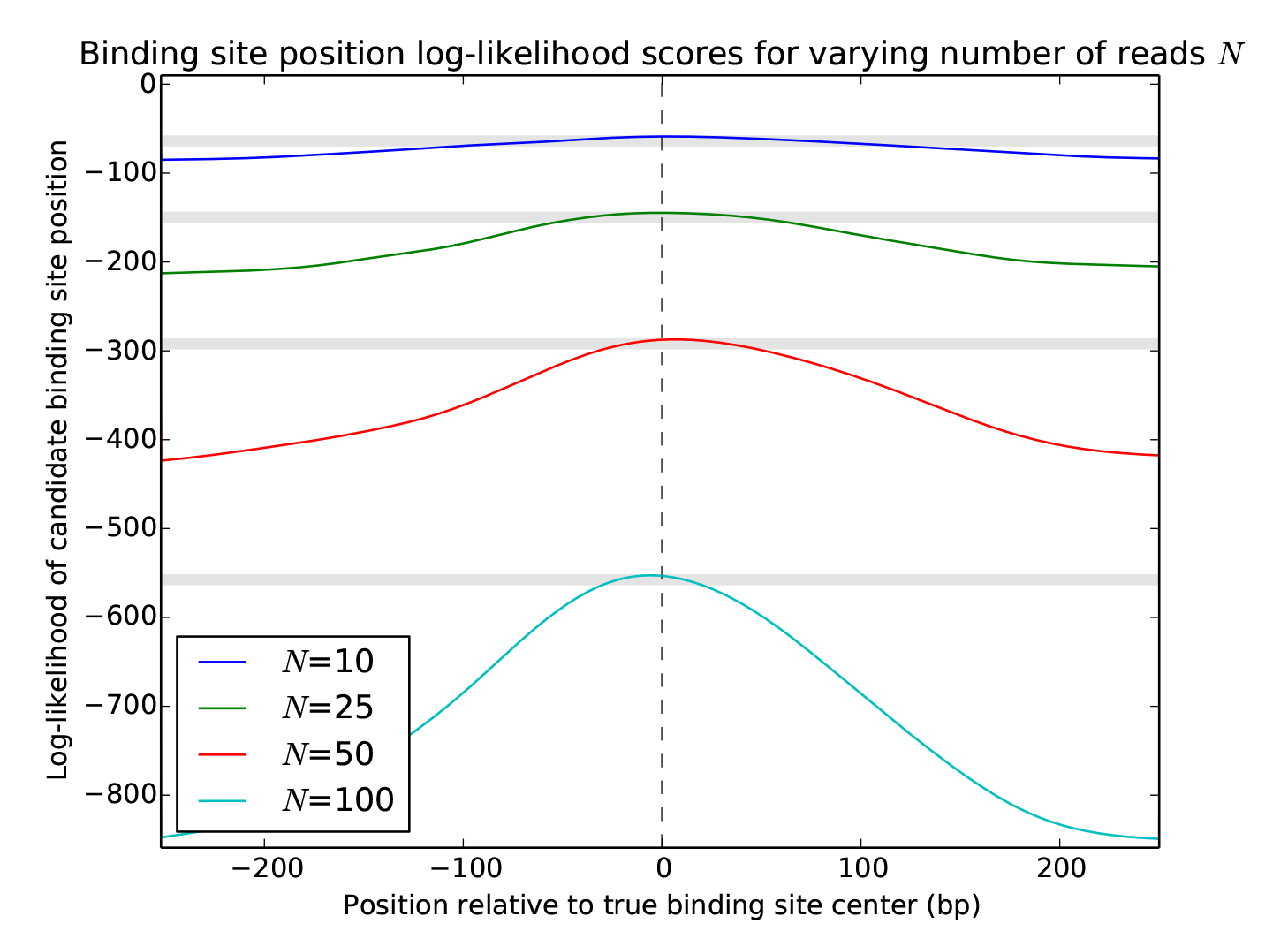

Supplement: Figure S7 — Log-likelihood assigned by MultiGPS to various positions around the optimal binding location, as a function of the number of reads associated with the binding event. Events with higher read counts have more sharply peaked log-likelihood landscapes, since there is more evidence pointing towards the optimal binding location. For illustration, we placed gray shaded bars representing a log-likelihood range of 10 around the peak of each log-likelihood distribution. The shaded bars illustrate the degree to which a typical cross-condition prior value (see Figure S6) can affect the binding location update step. If binding events are detected in nearby locations in each condition, the cross-condition prior will encourage them to align by overriding the optimal log-likelihood value found from read evidence alone. However, if events are associated with high read counts, the window in which the cross-condition prior can have an effect is reduced. This allows MultiGPS to detect truly distinct, but nearby located, condition-specific binding events if sufficient read evidence exists to support their existence in the model. (TIF) [file pcbi.1003501.s007.tif]
