# Supplementary material for: An Integrated Model of Multiple-Condition ChIP-Seq Data Reveals Predeterminants of Cdx2 Binding
Source: PLoS Comput Biol. 2014 Mar 27;10(3):e1003501. doi: 10.1371/journal.pcbi.1003501 (PMC3967921; doi:10.1371/journal.pcbi.1003501)
Supplement: Table S1 — MultiGPS increases sensitivity for detecting cell-type specific binding events. edgeR was used to do a three-condition analysis across the Tier 1 cell types for each protein, where each cell type/protein pair was performed twice. Recommended edgeR practices were used to estimate normalization factors and overdispersion amounts, and sites that had any condition-specific signature (testing for any of the coefficients in the regression model to be nonzero) were identified. The table reports the number of sites reported as significant by edgeR (FDR<0.01). (DOCX) [file pcbi.1003501.s008.docx]

| **Factor** | **MultiGPS cell-type specific sites** | **Windowed counts cell-type specific sites** | **MultiGPS pct. Increase** | **MultiGPS overdisp.** | **Windowed counts overdisp.** |
| --- | --- | --- | --- | --- | --- |
| ATF3 | 10426 | 5145 | 102.6 | 0.043 | 0.093 |
| CEBPB | 91628 | 72933 | 25.6 | 0.038 | 0.077 |
| CHD1 | 45680 | 18636 | 145.1 | 0.188 | 0.285 |
| CHD2 | 47840 | 39014 | 22.6 | 0.014 | 0.037 |
| CTCF | 60978 | 46338 | 31.6 | 0.035 | 0.053 |
| JUND | 73460 | 50161 | 46.4 | 0.023 | 0.063 |
| MAFK | 24128 | 11410 | 111.5 | 0.143 | 0.176 |
| NRSF | 69479 | 47595 | 46.0 | 0.033 | 0.067 |
| RAD21 | 129616 | 94976 | 36.5 | 0.053 | 0.104 |
| SIX5 | 10167 | 3205 | 217.2 | 0.067 | 0.130 |
| SP1 | 72623 | 58714 | 23.7 | 0.063 | 0.104 |
| SRF | 35261 | 16484 | 113.9 | 0.100 | 0.174 |
| TAF1 | 71227 | 49630 | 43.5 | 0.041 | 0.089 |
| TBP | 73442 | 61145 | 20.1 | 0.009 | 0.039 |
